# Pivotal Response Treatment (PRT) parent group training for young children with autism spectrum disorder: a pilot study

**DOI:** 10.1038/s41598-022-10604-2

**Published:** 2022-05-11

**Authors:** Manon W. P. de Korte, Martine van Dongen‑Boomsma, Iris J. Oosterling, Jan. K. Buitelaar, Wouter G. Staal

**Affiliations:** 1 Karakter Child and Adolescent Psychiatry University Centre, Nijmegen, The Netherlands; 2 Department of Cognitive Neuroscience, Donders Institute for Brain, Cognition and Behavior, Radboud University Medical Centre, Nijmegen, The Netherlands; 3 Leiden University, Leiden, The Netherlands

**Keywords:** Psychology and behaviour, Autism spectrum disorders

## Abstract

Pivotal Response Treatment (PRT) is a promising intervention addressing core symptoms of autism spectrum disorder (ASD), with parent involvement as key component. Parent group-delivered PRT may be an effective treatment model, but currently the evidence is limited. Also, little attention has been paid to therapeutic involvement of multiple important contexts (e.g. home, school, community) of the young child. The current study explores a 14-week protocol of PRT parent group training (PRT-PG), complemented with individual parent–child sessions and involvement of teachers and other childcare providers. Children aged 2–6 years old with ASD and their parents (*n* = 20) were included. Preliminary results showed a significant increase in spontaneous initiations during a semi-structured therapist-child interaction together with widespread gains in clinical global functioning. No significant improvement on parent-rated general social-communication skills was observed. These findings justify further research on parent group delivered PRT models.

Children with autism spectrum disorder (ASD) show impairments in the areas of social communication and interaction and present repetitive, restricted or non-compliant behavior, and sensory abnormalities^1^. The symptoms of ASD manifest in the early development of the child and may impact functioning throughout the individuals’ life^2^. Effective intervention approaches may alter the course of development and can have profound impact on functioning and well-being of young children with ASD and their families^3^. Since parents play a key role in the development of their child, parental involvement in treatment of young children with ASD is essential^4^. Training parents in several parent-mediated interventions may facilitate an intensive, naturalistic, and tailored approach, increasing a child’s progress of learned skills between environments^5^. Over the last 15 years, there has been rapid growth in research supporting the effectiveness of parent‐mediated interventions for improving language and communication, severity of autism symptoms, and parent–child interaction patterns of young children with ASD^6–8^.

Pivotal Response Treatment (PRT) is typically a parent-mediated intervention for children with ASD, aimed on targeting core areas (e.g. motivation and self-initiations) which in turn is hypothesized to facilitate widespread gains across multiple developmental domains^9,10^. Parents are taught to create learning opportunities in social communication for the child, according to the PRT principles; i.e. following the child’s interests, gaining the child’s attention, giving appropriate levels of help (prompting), providing immediate and contingent reinforcement in response to the child’s initiation or good attempt and interspersing maintenance and acquisition tasks^10,11^. The emphasis of parent training in PRT is on implementing these principles in the child’s daily life routines. In this manner, continuation of the intervention beyond the treatment setting and generalization of learned skills will be facilitated.

Findings from systematic reviews and recent randomized controlled trials on the efficacy of PRT indicate that parents are able to implement PRT techniques and that children with ASD show promising results regarding self-initiations, language development, general social-communication skills and collateral gains such as global functioning [e.g.^12–15^]. This body of research adds evidence on the efficacy of PRT to improve the core symptoms of ASD as well as on its potential to facilitate other domains of functioning by targeting social-communication skills. In addition, PRT parent training has also resulted in improved parent–child interactional patterns^16^ and decreased parental stress^17^. However, because of a high diversity in study methodology and implemented training models^18^, further research on PRT is warranted to strengthen and extend the evidence for the clinical value and implementation strategy of PRT.

In the last decade, converging evidence has emerged on parent group-delivered PRT models [e.g. 19,20,21]. In an uncontrolled trial of a 10-week during PRT parent group training (*n* = 17), the majority of the parents learned PRT at the end of the intervention and their children showed improvement in functional communication^19^. Also, significant gains in adaptive skills of children with ASD were found in a larger study (*n* = 158) with community-based PRT parent group training^22^. Furthermore, the effectiveness of parent group-delivered PRT and individual parent training (including parent–child sessions) on PRT were recently evaluated in a community-based treatment program^20^. Results indicated that – in contrast to individual parent training – parent group-delivered PRT resulted in gains in parental stress and self-efficacy. However, individual parent training resulted in large increases in spontaneous child initiations, whereas parent group training resulted in moderate increases. These findings suggest that an optimal treatment response may require a patient tailored PRT program in which parent group training is combined with individual sessions with a clinician.

At this point, only one study has examined the combination of a PRT parent group training with individual sessions with a clinician. In the randomized controlled trial of Hardan et al.^21^ Pivotal Response Treatment Group (PRTG)—consisting of eight parent-only sessions and four individual parent–child sessions—was compared to psychoeducation group (PEG) for 12 weeks. Compared to the PEG group, children in the PRTG demonstrated greater improvement in frequencies of verbal utterances and adaptive communication skills as measured by the Vineland-II^23^, and less severe social interaction and communication symptoms as measured by the Clinical Global Impression scale [CGI;^24^]. Further, in the follow-up evaluation of this trial it was found that positive effects of treatment were still present three months after the end of the intervention^25^. The findings of these studies support the combination of parent group training and individual parent–child sessions with a clinician as treatment approach. However, since these studies only targeted social communication domains (also on the CGI), little is known about the efficacy on more widespread gains such as global functioning and quality of life.

Besides the crucial role of parents, also other child care providers in the daily environment of the child may mediate the effects of PRT. For instance, many children with ASD have trouble in understanding or communicating their needs to educators and other children at school or the day care setting^26^. Teachers subsequently face challenges in understanding and managing the child’s behavior and may experience difficulties in creating an inclusive social environment within classrooms^27^. Recently, several studies have investigated the adaptation of PRT to the classroom context and reported that teachers are able to implement PRT techniques, resulting in positive outcomes on the child’s target goals [e.g.^28,29^]. Nevertheless, research to date has focused on only one setting (i.e. home or school) rather than integrating the whole social network of the child. In addition, other childcare providers—such as grandparents, babysitters etc.—can also have a profound role in the development of the child. Therefore, involvement of multiple contexts and childcare providers in PRT training of young children with ASD may improve the generalizability of learned skills in the natural environment.

Taken together, previous results on parent group delivered PRT are promising, but evidence is still limited. Additional research should explore the contribution of multiple contexts and evaluate effects on the child’s targeted skills and collateral gains in general mental health. Therefore, the primary goals of this pilot study were: (1) to design a PRT intervention protocol with parent group training, complemented with individual parent–child sessions and involvement of teachers and other childcare providers, and (2) to examine preliminary effects on core social-communication skills. In addition, collateral effects on severity of ASD symptoms, adaptive functioning, clinical global functioning, quality of life, and parenting stress were explored. This pilot study is the first step for successful follow-up investigation. Along with a (partial) parallel qualitative study on parental narratives, a more broaden evaluation on feasibility and treatment outcomes of this parent group PRT intervention protocol is established.

## Methods

### Design and participants

The current non-randomized pilot study was conducted at Karakter, a large academic hospital for child and adolescent psychiatry with several branches in the Netherlands. The study was approved by the institutional research committee of the treatment institute (22-8-2018). Participants were children (and their parents) who were referred for outpatient PRT intervention by a child psychiatrist or clinical psychologist of the department Karakter University Centre for Young Children, delivering highly specialized mental health care for children aged 0–6 years old. The inclusion criteria for children were: a) 2–6 years old, b) clinical diagnosis of ASD, based on thoroughly diagnostic assessment by a multidisciplinary expert team with a child and adolescent psychiatrist or clinical psychologist and according to DSM-5 criteria^1^, c) ability to speak with single words at minimum (based on observation and parent report). The inclusion criteria for the parents were that: a) they did not receive PRT training before, b) they were willing to participate in parent group sessions, c) at least one parent was available to consistently attend the sessions, d) they spoke Dutch. Participants were not excluded if they received concomitant outpatient interventions such as pharmacotherapy or parent-guidance on parenting skills. All procedures performed in this study were in accordance with ethical standards of the institutional and national research committee and with the 1964 Helsinki declaration and its later amendments or comparable ethical standards.

### Procedures

Recruitment of the participants took place between 2018 and 2020. Parents received information on the aim of the study and they were evaluated on eligibility criteria. After agreement on participation, written informed consent was obtained from all parents. If parents did not want to participate in the study, they had the possibility to receive individual PRT training. Group enrolment was capped at four families per group, led by two certified PRT therapists (level III). In total, four certified PRT therapists were involved in the study. Meetings with PRT therapists and supervision meetings with a certified PRT-trainer (level V) were held for additional training and supervision. Each family was linked to one therapist that provided the individual sessions. After approvement of the parents, the PRT therapist informed the child’s teachers about procedures of the intervention protocol. Before baseline measures, all parents received psychoeducation on ASD to improve knowledge about their child’s behavior. All measurements were taken before the start of the intervention (baseline) and directly after the intervention (endpoint).

### Pivotal Response Treatment Parent Group training (PRT-PG)

The PRT-PG intervention was based on the PRT guidelines^30,31^ and was focused on improving social-communication skills. Before the implementation of the current study, a pilot investigation of the PRT-PG protocol was performed as a trial run. Specifically, one treatment group consisting of four children and their parents followed the PRT-PG intervention, led by two PRT therapists. Pilot testing facilitated the examination of intensity and frequency of sessions, (use of) video upload systems, course material and video examples etc. Suggestions of the therapists and parents were related to the timing and order of the sessions (i.e. teacher session two weeks earlier and switch of some individual and group sessions), and on (translation of) phrases in the post-treatment parent survey. Based on these suggestions, minor adjustments were accomplished for the final treatment protocol.

The intervention model of PRT-PG consisted of 14 weekly sessions. Of the 14 sessions, five were 90-min parent group sessions, four were 45-min individual parent–child sessions, two were 45-min individual parent-only sessions, one was a 90-min individual teacher session and there was one 90-min social network group session. Ten weeks after the end of the intervention there was a 90-min follow-up parent group session. Specific content of the sessions is outlined in Supplement 1. Treatment goals were based on parent and teacher reports and clinical observation during the first individual parent–child session. Goals were mainly focused on aspects of spontaneous functional self-initiations in the communication (e.g. expanding length of utterance, asking for an object/activity, asking for help, commenting) and the goals were adjusted to the child’s needs. During the parent group sessions, parents received background information on PRT and explanation of the motivational PRT techniques (i.e. following child’s choice, gaining the child’s attention, providing clear learning opportunities, direct and natural reinforcement and rewarding attempts, and interspersing maintenance and acquisition tasks). Presenting course material and video examples were used to illustrate. Parents were also given homework, consisting of practicing and video recording the implementation of the PRT techniques during parent–child interaction in the home setting. Specifically, they were instructed to practice with their child during (1) a game play activity and (2) activity in the daily routine. In the next group session, these videotaped interactions in the home setting along with the videotaped parent–child dyads of the individual sessions were discussed. Parents were encouraged to give each other positive performance-based feedback on the implementation of the PRT techniques. The therapists provided suggestions and additional information if needed. During the individual parent–child sessions the therapist of the family demonstrated the PRT techniques during interaction with the child and the therapist coached parents in applying the techniques during parent–child interaction (e.g. playing a game, physical activity, drawing etc.). These sessions were recorded on video for later review. In the individual parent only sessions the therapist evaluated the goals and progress of the parents and the child. During the individual teacher session, the therapist informed and trained the child’s teacher in the use of PRT techniques at school. In the social network group session, all parents in the group had the possibility to bring along other important childcare providers in the development of their child, such as grandparents, other family members, host parents or babysitters. The motivational PRT techniques were explained and illustrated using parent–child video recordings of the participants. Target goals of the participating children of the group were discussed and childcare providers could ask questions about the adaption of PRT in specific situations.

## Measures

### Demographics

Demographic data of children (i.e. age, sex, total intelligence quotient (TIQ), psychiatric comorbidity, and medication use) and parents (i.e. age, educational level) were gathered from electronic case files of Karakter.

### Outcome measures

General social-communication skills (i.e. skills that the child shows in his/her natural environment) were measured using the Social Responsiveness Scale—Second Edition [SRS-2;^32^]. The 65-item digitalized questionnaire, rated on a 4-point scale, was filled in by parents. The SRS-2 scale has excellent test–retest reliability (0.88-0.95) and an interrater reliability of (0.61-0.92) and good internal consistency (0.95)^32^. Raw total scores and subscale scores (i.e. Social communication, Social cognition, Social consciousness, Social motivation, and Autistic preoccupations) were computed, with higher scores representing more impairment in general social-communication skills.

Gains in social-communication skills of the child were also assessed during a semi-structured therapist-child interaction, using a rating scheme (see Supplement 2). Baseline assessments were done by the PRT therapist of the family at the first individual parent–child session. To account for potential bias, endpoint assessments were held by an independent PRT therapist who was unfamiliar with the child. The PRT therapist elicited learning opportunities for the following operational defined communication skills: one-word utterance, two-word utterances, asking for an object/activity, asking for help, wh-question asking (e.g. what, where, which, when), protesting, interrogating, making statements and responding to multiple cues. Percentage of spontaneous (without prompting) initiations was calculated dividing the number of therapist-elicited learning opportunities in which the child showed contextually appropriate initiations without provided verbal prompts by the total number of therapist-elicited learning opportunities.

Severity of ASD-related symptoms were assessed using the Dutch version of the Autism Diagnostic Observation Scale—Second edition (ADOS-2;^33^). Module 1, 2 or 3 was administered, according to age and level of spoken language. The ADOS-2 manual reports fair to excellent internal consistency (0.60–0.95), good interrater reliability (0.90–0.96) and fair to good test–retest reliability (0.64–0.92)^34^. In the current study the ADOS was administered and coded by psychologists trained up to research reliability.

The ADOS-2 was performed by a certified clinician who was unfamiliar with the participant and earlier outcomes on the ADOS-2. Raw total scores were used as also subdomain scores for the Social Affect (SA) and Restricted and Repetitive Behavior (RRB) domains. In addition, Calibrated Severity Scores [CSS;^35^] were calculated, with higher scores indicating more severe ASD symptoms.

Adaptive functioning was assessed using the digitalized Vineland screener 0–12 years research version^36^, consisting of 90 items. Available data on the Vineland screener support the reliability and validity of the instrument. Internal consistency of the domains and test–retest reliability are high: 0.90 or more^36^. Items were arranged according to the following subscales: Communication, Daily Living Skills, Socialization, Motor Skills. Also, the Adaptive Behavior Composite score was calculated. Higher scores represent higher adaptive behavior.

The CGI questionnaire was used for assessment of clinical global functioning, consisting of an severity score (CGI-S) and improvement score (CGI-I). There is no information on the validity and reliability of the CGI. The questionnaire was rated on a 7-point scale (CGI-S; *normal—among the most extremely ill patient,* CGI-I; *very much improved—very much worse*) by an experienced independent child- and adolescent psychiatrist who was unfamiliar with the participant. In total, two child- and adolescent psychiatrists were involved in this study. The psychiatrist based their rating on information about clinical status of overall functioning, symptoms, and well-being in major areas of the participants life (i.e. home, school/day care). Clinical responders were defined as children that were rated *very much improved* (score 1) or *much improved* (score 2).

Quality of Life (QoL) of the child was measured using a 10-point rating scale (*very low-very high* ), rated by the parent.

Parenting stress was assessed, using the digitalized 34-items using the Dutch Parenting Stress Questionnaire “Opvoedingsbelastingvragenlijst” [OBVL;^37^]. The OBVL contains 5 scales: Problems in parenting, Problems in parent–child relation, Depressive mood, Role-restriction, and Health complaints. Parents rated whether the items were applicable to them on a 4-point scale. Total T-scores were computed and used for analyses, with higher scores indicating more parenting stress.

### Post-treatment parent survey

In order to evaluate the PRT-PG intervention and to get insight in acceptability and feasibility, a post treatment questionnaire was administered by parents. The questionnaire was based on the survey as reported in the study of Bradshaw^38^, with minor adjustments for an appropriate translation in Dutch. The questionnaire consists of 20 statements, using a 5-point scale (*strongly disagree*-*strongly agree*). The statements were related to three global themes: Satisfaction with the intervention (8 items), Observed gains (5 items), Parental stress and concern (7 items). Parents were asked to administer this questionnaire at the last group session.

### Statistical analyses

To explore the effectiveness of PRT-PG , statistical analyses were performed with SPSS Statistics^39^ with a significance level set at *p* = 0.05. Distribution of normality was checked for all variables. There were no outliers (z-cores + /- 3). Paired samples t-tests were performed to examine changes in continuous outcome measures from pre- to post-intervention. Effect sizes were calculated using Cohen’s *d*, with 0.2, 0.5 and 0.8 representing small, medium and large effect sizes, respectively^40^. In the context of this exploratory study, no correction for multiple testing was performed.

## Results

### Study population

In total, 27 participants initiated the PRT-PG intervention during the period of February 2018-June 2020. However, because of the impact of the COVID-19 pandemic and restrictions to have face-to-face sessions, the last seven participants (two groups) could not adhere to the intervention protocol. Five participants continued the intervention via videoconferencing, but protocol adjustments were necessary according to individual needs and possibilities. Therefore, these last seven participants were excluded from analyses in the current study, resulting in a total sample of 20 participants (distributed over five groups). One of these 20 participants discontinued the intervention protocol after seven sessions, due to family related factors. However, all 20 participants completed the assessments. See Table 1 for demographic characteristics. Parents ranged in age from 27 to 52 years, with a mean age of 39.79 years (SD = 6.18) for fathers and a mean age of 36.05 years (SD = 5.03) for mothers. In half of the participants only mothers attended the sessions (*n* = 10) and in the other half of the participants both parents participated (*n* = 10). The parents of the participants generally had a degree in intermediate vocational education, or higher professional education or university.

**Table 1:** Demographic characteristics.

	*N* = 20	
	Mean/*n*	(SD/%)
Age in years (range 3–7)	4.92	(1.07)
**Sex**		
Male	18	(90%)
Female	2	(10%)
TIQ	91.93	(16.80)
**Psychiatric comorbidity**		
AD(H)D	2	(10%)
Other	2	(10%)
**Medication use**		
Stimulants	2	(10%)
Antipsychotics	0	(0%)
Age in years mother (range 23–52)	35.85	(4.58)
Age in years father (range 27–48)	40.08	(6.20)
**Level of education mother**		
Primary/ secondary	0	(0%)
Vocational/advanced	9	(45%)
Bachelor’s degree or higher	10	(50%)
Unknown	1	(5%)
**Level of education father**		
Primary/secondary	1	(5%)
Vocational/advanced	8	(40%)
Bachelor’s degree or higher	9	(45%)
Unknown	2	(10%)

*AD(H)D*, Attention deficit (hyperactivity) disorder; *TIQ*, total intelligence quotient.

### Outcome measures

The total scores and subscale scores on the parent-rated SRS did not differ significantly between baseline and endpoint (see Table 2), indicating no significant improvement in general social-communication skills. However, a trend towards significance was observed for the SRS motivation subscale score, representing less impairment in social motivation at the end of the intervention (*t*(19) = 1.97, *p* = 0.06, *d* = 0.42). Regarding social-communication skills of the child during the semi-structured therapist-child interaction, there was a significant change from baseline to endpoint observed for the percentage of spontaneous initiations (*t* = -3.45 (14), *p* = 0.004, *d* = 0.86). There were no significant changes from baseline to endpoint for any of the ADOS-2 and Vineland-screeners outcomes. There was also no significant change in parent-rated quality of life (QoL) of the child. The results of the OBVL total t-score, showed a trend towards lower perceived parenting stress at the end of the intervention (*M* = 57.90, *SD* = 10.18*)* compared to baseline (*M* = 60.70, *SD* = 8.36*), t* = 1.00 (19), *p* = 0.067, *d* = 0.30. Pre-post analyses on the independently rated CGI established a significant decrease in the severity score (*t* = 2.70 (19), *p* = 0.01, *d* = 0.69), indicating less severe clinical symptoms in global functioning at the end of the PRT-PG intervention. The CGI improvement score (CGI-I) revealed improvement in global functioning for 80.0% of the participants (score 1,2 or 3), with a clinical responder (score 1 or 2) rate of 22.2%.

**Table 2:** Baseline and endpoint outcome comparisons. Vineland ABC score: Vineland screener 0-12 research version Adaptive Behavior Composite score.

	Baseline (n = 20)		Endpoint (n = 20)		Baseline to endpoint		
	M (SD)		M (SD)		*t* (df)	*p*	*d*
**SRS parents**							
Total score	78.50	(18.10)	75.05	(18.59)	1.27 (19)	.221	0.19
Communication	24.10	(6.18)	23.90	(7.23)	0.14 (19)	.888	0.03
Cognition	16.50	(4.10)	15.50	(4.67)	1.26 (19)	.222	0.23
Consciousness	9.95	(2.82)	9.95	(1.91)	0.00 (19)	1.00	0.00
Motivation	13.40	(3.27)	12.00	(3.31)	1.97 (19)	**.064**	0.42
Preoccupations	14.55	(4.82)	13.70	(5.36)	1.02 (19)	.323	0.17
Initiations *							
% Spontaneous	33.33	(18.85)	50.83	(21.63)	−3.45 (14)	**.004**	0.86
**CGI**							
Severity score	5.15	(0.64)	4.55	(1.05)	2.70 (19)	.010**	0.69
Improvement score	−		2.90	(0.91)			
Clinical responder	−		22.20	%			
**ADOS-2**							
Total score	10.58	(3.67)	10.37	(5.21)	0.25 (18)	.803	0.05
Social affect	8.68	(3.70)	8.53	(4.95)	0.18 (18)	.853	0.03
Repetitive behavior	2.37	(2.52)	1.63	(1.30)	1.04 (18)	.312	0.37
CSS	5.68	(1.38)	5.47	(2.34)	0.44 (18)	.663	0.11
**OBVL**							
Total (T-)score	60.70	(8.36)	57.90	(10.18)	1.99 (19)	**.067**	0.30
**Vineland screener**							
ABC	95.65	(21.74)	99.25	(22.69)	−1.90 (19)	.073	0.16
Communication	22.65	(7.80)	23.65	(7.42)	−1.78 (19)	.091	0.13
Socialization	24.10	(7.26)	25.15	(7.13)	−1.01 (19)	.326	0.15
Daily living skills	21.25	(6.48)	22.60	(7.25)	−1.62 (19)	.121	0.20
Motor skills	27.65	(4.04)	27.85	(4.58)	−0.26 (19)	.798	0.05
Quality of life	8.07	(0.73)	7.86	(1.17)	1.39 (13)	.189	0.22

* As response on therapist-elicited learning opportunities. ^a^ Score 1 (very much improved) or score 2 (much improved) on CGI-I. Significance values are in [bold].

### Post-treatment parent survey

The evaluation survey was administered by 23 parents at the end of the intervention. The items were collapsed in three categories; Satisfaction with the intervention, Observed gains, Parental stress and anxiety. Overall, all parents were satisfied with the intervention as indicated by a 4 out of 5 rating on the *Satisfied with Intervention* items (SD = 0.33). More specifically, all parents agreed (score 4 or 5) that they learned strategies to help their child to communicate effectively and that they used the PRT techniques outside the sessions. Furthermore, a large majority of parents agreed that they learned strategies to have social interaction with their child (91%) and that they were more confident helping their child to socially engage (87%) and to communicate (87%). More than half of the parents stated that the intervention was easy to implement outside the sessions (64%) and that the intervention improved their own and their child’s daily life (57%). The overall rate on *Observed gains* was 3.85 out of 5 (SD = 0.54). Most of the parents observed gains in their child’s communication (83%) and social engagement (83%) and most of the parents agreed that their child communicates more efficiently at the end of the intervention (81.8%). However, only 45% of the parents stated that their child is more social. In total, 91% of the parents stated that they have benefited from the intervention. The overall rate on the *Parental stress and concern* category was 2.53 out of 5 (SD = 0.35), with diffused responses. About half of the parents reported that they were concerned about the development of their child before the intervention (57%). In total, 48% of the parents reported that the intervention decreased their concerns and feelings of stress about their child. None of the parents reported that they had more concerns about (the development of) their child after the intervention, but one parent (4%) noted that the intervention raised feelings of stress about their child.

## Discussion

The aim of the current pilot study was to design and to evaluate a parent group delivered PRT protocol, incorporating the most important environments for the child (i.e. home, school/day care, community). This study was the first to investigate PRT parent group training (PRT-PG), complemented with individual parent–child sessions and involvement of teachers and other childcare providers. Effects on social-communication skills were explored, along with more widespread effects of treatment on ASD symptom severity, adaptive functioning, clinical global functioning, quality of life, and parenting stress.

Almost all participants included in this study strictly adhered to the 14-week intervention protocol; only one participant discontinued the intervention due to family related factors. The results showed that the PRT-PG intervention did not lead to significant improvement in parent-rated general social-communication skills. However, after 14 weeks a significant increase in therapist-elicited spontaneous initiations was observed during a semi-structured therapist-child interaction. There was no significant progress at the end of the intervention in children’s ASD symptom severity, adaptive functioning, or quality of life, but a trend was found in decreasing parenting stress. The results of the independent rated CGI showed a significant decrease in severity of clinical symptoms in global functioning at the end of the PRT-PG intervention. Furthermore, the findings of the post treatment questionnaire showed that a large majority of the parents learned strategies to promote social-communication skills and that most parents observed gains in the child’s communication skills and social engagement. Taken together, parents seem to be able to learn and implement PRT strategies during PRT-PG, but results regarding the effects on child’s social-communication skills and collateral gains are diffused.

The current findings on general social-communication skills contradict previous research on PRT that have reported significant improvement on the SRS as outcome measure^14,41,42^. However, the results are in line with a comparable prior study of Hardan et al.^21^ on parent group delivered PRT (complemented with individual parent–child sessions) that also did not found significant treatment effects on SRS total raw scores^21^. A possible explanation of this discrepancy in results of PRT research is the main focus on parent training and a relatively short duration of the intervention protocol (i.e. 12–14 weeks) in the current study and the study of Hardan et al.^21^. This short duration may not lead to clinical changes in child’s general social-communication skills directly after treatment but suggests a ‘sleeper effect’ : more time is needed to result in larger benefits^43^. Instead, improvements in social-communication skills may be observed in more proximal observational measures such as parent–child lab observation^21^ or semi-structured therapist-child interaction as reported in this study. Nevertheless, it is interesting that results of the post-treatment survey showed that most parents reported gains in their child’s communication (90%) and social engagement (85%). Therefore, the use of the SRS as outcome measure for change in general social-communication skills in (PRT) intervention research could be reconsidered^44^ .

As PRT is hypothesized to not only target core ASD symptoms (i.e. social-communication skills) but also collateral gains on the development of the child, this study included measures on widespread child and parent outcomes. The results on the independent clinician rated CGI indicated less severe clinical symptoms in global functioning at the end of the intervention, which is in line with previous findings^15,21,45,46^. Also, the improvement rating of the CGI (CGI-I) demonstrated that 80% of the participants showed improvement in global functioning, of which 22% of the participants represented a substantial change and could be indicated as a clinical responder (*very much improved* or *much improved*). This overall positive but subtle change might be related to the relatively low intensity and duration of the intervention and to directly post-treatment assessments. More time and additional services might be needed to observe clinically meaningful improvement in global functioning in a population as examined in this study (i.e. highly specialized mental health care).

In contrast to expectations based on previous research^21,22^, the current study could not support the hypothesis that PRT leads to collateral improvement in adaptive functioning. Furthermore, there were no changes in quality of life ratings. It is interesting though that the parents’ baseline and endpoint perspectives on quality of life of their child were both around 8 out of 10, with a common explanation that their child is happy, cheerful and healthy. Since this study is the first in examining (change in) quality of life in children with ASD receiving PRT, further research on the concept of quality of life in PRT intervention studies is suggested.

Lastly, the results showed that after the intervention there was no significant decrease in parenting stress, which is in contrast to findings of Verschuur et al.^20^ and Minjarez et al.^17^ on PRT parent group training. However, about half of the parents did note on the post survey questionnaire that the intervention decreased their concerns and stress about their child. Since parenting stress might not only be attributable to the child’ social-communication deficits but can also be related to the overall impact of having a child with ASD on their life and well-being^47^, more insight is needed in the origin and the content of the stress parents experience. Moreover, the added value of a group context on parents’ learning capacity and well-being should be further explored.

Results on the post survey questionnaire revealed that the large majority of the parents (91%) were positive about the intervention and benefited from the program. More specifically, all parents agreed that they learned strategies to help their child to communicate effectively and that they used the PRT techniques outside the sessions. In addition, more than half of the parents stated that the intervention was easy to implement outside the sessions and that the intervention improved the daily life of themselves and their child . These results suggest that in general the PRT-PG intervention is satisfactory to parents. In addition, there was a very low drop-out rate, indicating feasibility and acceptability of the intervention. It should be noted though that this study included parents who were willing to participate in a group setting. Therefore, generalization of findings should be interpreted with some caution.

This pilot study included different kind of outcome measures to identify treatment gains; i.e. observations during a semi-structured therapist-child interaction, parent reports, and independent clinician ratings and observations. The combination of these proximal (specific to the treatment targets) and distal measures, as also standardized objective assessments, strengths the methodology of this study. However, a limitation was that due to the exploratory character of this study, some low time-consuming screening instruments were chosen rather than expanded interviews and questionnaires. For instance, the Vineland-screener research version (0–12 years old) was used compared to the expanded Vineland-II survey version in previous research on PRT for young children with ASD (i.e.^21^). Further, this study included a simplistic rating scale (score 0–10) for measuring quality of life (QoL) of the child—as estimated through the vision of the parent—rather than a parameter such as the PedsQL^48^. Therefore, future research with standardized tools are warranted to strengthen the knowledge of beneficial effects on children and parents after PRT.

Several aspects limit the interpretation of the current study. The sample size was small and there was no control group included, with the consequence of low statistical power. Further, we did not include the examination of fidelity of implementation and there was no insight in the continuation of PRT implementation and perceived effects after the intervention period of the study. Lastly, ratings were mainly based on parent’s perspectives and there were no fully blinded outcome measures. To avoid observer bias, future research should include multiple (blinded and non-blinded) informants (e.g. teachers, parents, clinicians). As the primary goal of the current study was to design and explore effects of a new PRT parent group training intervention protocol, the current study should be interpreted as preliminary findings and cannot be considered as conclusive. A (partial) parallel qualitiative study on this intervention protocol will allow an in-depth examination of parent’s perspectives on the value of a parent group, individual sessions and the involvement of multiple contexts. Also, parental narratives on perceived effects will be explored. Future research including an experimental design with independent measures, larger sample size, and follow-up period are warranted for further examination of this intervention protocol.

Taken together, the findings of this pilot study suggest initial feasibility and efficacy of PRT parent group training complemented with individual parent–child sessions and involvement of multiple contexts on child’s social-communication skills during therapist-child interaction and global functioning. Qualitative reports may help to further understand the value of different aspects of the intervention protocol and the effects on both child and parental outcomes. In addition, large-scale experimental research is warranted to further understand the implications of PRT parent group training and to optimize individual outcomes.

## Supplementary Information

Supplementary Information 1.

Supplementary Information 2.
