# Supplementary material for: Pivotal Response Treatment (PRT) parent group training for young children with autism spectrum disorder: a pilot study
Source: Sci Rep. 2022 May 11;12:7691. doi: 10.1038/s41598-022-10604-2 (PMC9095862; doi:10.1038/s41598-022-10604-2)
Supplement: Supplementary file 1 — Supplementary Information 1. [file 41598_2022_10604_MOESM1_ESM.docx]

| **Table S1.** Overview of PRT-PG content | | |
| --- | --- | --- |
|  | Type | Content |
| 1 | Individual parent | - Overview PRT protocol - Discussing treatment goals and expectations |
| 2 | Individual  parent-child | - Semi-structured therapist-child interaction - Practicing PRT during parent-child interaction |
| 3 | Group | - Theoretical background PRT + overview techniques - In-depth illustration of 1) following the child’s interest and 2) gaining the child’s attention - Review on video’s session 2 |
| 4 | Individual parent-child | - Practicing PRT during therapist-child & parent-child interaction |
| 5 | Group | - Review on home videos of parents |
| 6 | Group | - In-depth illustration of step 3) creating clear learning opportunity and use of prompts and 4) natural, immediate and contingent reinforcement and rewarding attempts - Review on home videos |
| 7 | Individual parent-child | - Practicing PRT during therapist-child & parent-child interaction |
| 8 | Individual parent | - Discussing parent and child progress - Evaluation of target goals |
| 9 | Individual teacher | - Observation in classroom - Instruction of PRT to teacher |
| 10 | Group | - Review on home videos - Discussion on maintenance/acquisition of tasks, task variation and self-management |
| 11 | Individual parent-child | - Semi-structured therapist-child interaction - Practicing PRT during parent-child interaction |
| 12 | Social network Group | - Presence of other individual child care providers in parent group - Overview PRT and illustration based on videos (from home or individual session) |
| 13 | Group | - Review on home videos of parents - Discussion on future goals |
| 14 | Individual parent | - Evaluation with the family’s child psychologist and the PRT therapist |
|  |  |  |
| 15 | Group  (follow-up) | - Review on home videos and experiences - Group evaluation |
|  |  |  |
|  |  |  |
