# Supplementary material for: Pivotal Response Treatment (PRT) parent group training for young children with autism spectrum disorder: a pilot study
Source: Sci Rep. 2022 May 11;12:7691. doi: 10.1038/s41598-022-10604-2 (PMC9095862; doi:10.1038/s41598-022-10604-2)
Supplement: Supplementary file 2 — Supplementary Information 2. [file 41598_2022_10604_MOESM2_ESM.docx]

|  | **Initiation without verbal prompt** | | **Initiation with verbal prompt*** | | |  |
| --- | --- | --- | --- | --- | --- | --- |
|  | **Spontaneous** | **Waiting** | **Open question prompt** | **Fill-in prompt** | **Tell prompt** | **No Response** |
|  |  |  |  |  |  |  |
| One-word utterance |  |  |  |  |  |  |
| Two-word utterances |  |  |  |  |  |  |
| Asking for an object/activity |  |  |  |  |  |  |
| Multiple cues |  |  |  |  |  |  |
| Asking for help |  |  |  |  |  |  |
| Wh-question asking (e.g. what, where, which, when) |  |  |  |  |  |  |
| Protesting |  |  |  |  |  |  |
| Interrogating |  |  |  |  |  |  |
| Making statements |  |  |  |  |  |  |

**Supplement S2.** Coding scheme of operational defined social communication skills, assessed during a semi-structured therapist-child interaction.

* Examples of verbal prompt:

- Open question prompt: “What can you ask me now?”
- Fill-in question prompt: “Can I have…..”
- Tell prompt: “You can say: Can I have the ball?”
